# The Buzz about ADP-Ribosylation Toxins from *Paenibacillus larvae*, the Causative Agent of American Foulbrood in Honey Bees

**DOI:** 10.3390/toxins13020151

**Published:** 2021-02-16

**Authors:** Julia Ebeling, Anne Fünfhaus, Elke Genersch

**Affiliations:** 1 Department of Molecular Microbiology and Bee Diseases, Institute for Bee Research, 16540 Hohen Neuendorf, Germany; julia.ebeling@hu-berlin.de (J.E.); anne.fuenfhaus@hu-berlin.de (A.F.); 2 Department of Veterinary Medicine, Institute of Microbiology and Epizootics, Freie Universität Berlin, 14163 Berlin, Germany

**Keywords:** ADP-ribosylation, bacterial toxins, American Foulbrood, *Paenibacillus larvae*, honey bee disease

## Abstract

The Gram-positive, spore-forming bacterium *Paenibacillus larvae* is the etiological agent of American Foulbrood, a highly contagious and often fatal honey bee brood disease. The species *P. larvae* comprises five so-called ERIC-genotypes which differ in virulence and pathogenesis strategies. In the past two decades, the identification and characterization of several *P. larvae* virulence factors have led to considerable progress in understanding the molecular basis of pathogen-host-interactions during *P. larvae* infections. Among these virulence factors are three ADP-ribosylating AB-toxins, Plx1, Plx2, and C3larvin. Plx1 is a phage-born toxin highly homologous to the pierisin-like AB-toxins expressed by the whites-and-yellows family *Pieridae* (Lepidoptera, Insecta) and to scabin expressed by the plant pathogen *Streptomyces scabiei*. These toxins ADP-ribosylate DNA and thus induce apoptosis. While the presumed cellular target of Plx1 still awaits final experimental proof, the classification of the A subunits of the binary AB-toxins Plx2 and C3larvin as typical C3-like toxins, which ADP-ribosylate Rho-proteins, has been confirmed experimentally. Normally, C3-exoenzymes do not occur together with a B subunit partner, but as single domain toxins. Interestingly, the B subunits of the two *P. larvae* C3-like toxins are homologous to the B-subunits of C2-like toxins with striking structural similarity to the PA-63 protomer of *Bacillus anthracis*.

## 1. Introduction

Pathogenic bacteria cause disease by infecting their eukaryotic hosts and then exploiting them for proliferation and transmission. Causing disease, however, is rather not the “objective” of pathogenic bacteria, but can in most cases be viewed as collateral damage as the bacteria attempt to achieve their primary goals, proliferation, and transmission. In order to successfully infect their hosts, pathogenic bacteria use virulence factors that enable and control the various steps in pathogenesis, such as adherence to host cells, invasion of the host by breaching cellular barriers, multiplication within the host, and evasion of host defenses. An important group among these virulence factors are the bacterial protein toxins (exotoxins), which are ancient pathogen weapons used to manipulate host cell functions to enable infection. Most exotoxins are characterized by a very specific activity that is restricted to certain cell types and depends on the interaction of the toxins with certain cell membrane receptors. Exotoxins can be grouped into three categories based on their mode of action (membrane acting, membrane damaging, intracellular toxins). Within each of these categories, the toxins can be further subdivided based on their enzymatic activity. One of the enzymatic activities carried out by intracellular toxins is the covalent conjugation of ADP-ribose derived from NAD^+^ to intracellular acceptor molecules. This ADP-ribosylation leads to the loss of function of the targeted molecules and subsequent deregulation of key cellular processes.

The first bacterial mono-ADP-ribosyltransferase (ART) toxin to be identified was the diphtheria toxin produced by *Corynebacterium diphtheria* [1,2]. Thereafter it became evident that ADP-ribosylation is an enzymatic reaction that is often used by bacterial toxins to inactivate or manipulate target molecules from host cells. Given their widespread occurrence in bacterial pathogens, it is not surprising that ART toxins also play a role in the pathogenesis of infections caused by *Paenibacillus larvae*, an entomopathogenic, Gram-positive, spore-forming bacterium.

*P. larvae* is the etiological agent of American Foulbrood (AFB), a highly contagious and often fatal bacterial disease of the brood of the Western honey bee, *Apis mellifera* [3]. AFB is a notifiable disease in most countries and no sustainable control measures for this devastating disease are known. *P. larvae* and the associated AFB are globally distributed. Most if not all worldwide cases of AFB are caused by *P. larvae* strains belonging to two different genotypes, ERIC I and ERIC II, which can be distinguished via repetitive element polymerase chain reaction (rep-PCR) performed with primers amplifying enterobacterial repetitive intergenic consensus (ERIC) sequences [3,4]. These two genotypes differ in virulence at individual larva- [5] and colony-level [6] as well as in their pathogenesis strategies during the invasive phase [7]. The *P. larvae* ERIC genotypes III–V have not been isolated from AFB outbreaks over decades, but only exist as historical isolates in culture collections (ERIC III/IV) [3] or have been isolated as single strains from honey [8,9]. Representatives of these three genotypes are characterized by hypervirulence [3,8,9], which could be responsible for their failure to establish themselves as pathogens in the honey bee population.

Infection starts when young larvae take up food contaminated with spores of *P. larvae*. Ingested spores germinate in the midgut lumen, where the vegetative bacteria then multiply massively without damaging the epithelium [10]. This commensal-like phase, as well as the beginning of the invasive phase when the chitin-degrading enzyme *Pl*CBP49 breaks down the peritrophic matrix protecting the epithelium [11], is the same for both genotypes. Consequently, *Pl*CBP49 has been identified as a key virulence factor for the species *P. larvae* [12]. The subsequent breaching of the defenseless epithelial barrier and penetration into the larval hemocoel, however, proceeds differently for *P. larvae* ERIC I and ERIC II because the genotypes differ in the cocktail of virulence factors expressed and used during this phase [7,13]. Only *P. larvae* ERIC II expresses the S-layer protein SplA [14], which mediates bacterial attachment to the epithelial cell layer as the first step in attacking and breaching the epithelium [15]. In contrast, *P. larvae* ERIC I does not adhere to the epithelium but instead uses toxins to attack and destroy the midgut epithelium [7,13,16]. Invasion of the larval hemocoel coincides with larval death [10], which in turn initiates the final, saprophytic phase of *P. larvae* pathogenesis. During this phase, the larval cadaver is degraded to a glue-like mass, which dries out to a highly infectious scale consisting of millions of newly generated *P. larvae* spores. No virulence factors are known so far for this pathogenesis phase, but siderophores and antibiotics produced by *P. larvae* most likely play a role in preventing saprophytic microbial competitors from taking over the larval remains [17,18,19,20].

Comparative genome analysis has identified seven and three putative toxin loci in the genomes of *P. larvae* ERIC I and ERIC II, respectively [7,13]. All of them showed similarity to the family of AB toxins and contained ORFs with similarity to ADP-ribosylation domains and binding/translocation domains. However, a closer look revealed that the three loci in *P. larvae* ERIC II were interrupted by mutations and transposases, and therefore, functional toxin production in *P. larvae* ERIC II was excluded [7]. In *P. larvae* ERIC I, the already identified and experimentally characterized AB-toxins Plx1 and Plx2 [16] were confirmed, and three other putatively functional AB toxin loci, Plx3-5, were identified [7], but their activity and biological role still await experimental proof. The other two AB toxin loci, designated Tx6 and Tx7, were considered non-functional due to inserted transposases and mutations splitting the genes coding the putative A or B domains into several ORFs [7]. Recently, the *P. larvae* toxin C3larvinAB has been described [21], which is only functional in a unique strain of *P. larvae* [9,22], which is the only representative of the MLST sequence type 9 within *P. larvae* ERIC III/IV [4]. In all other genotypes/sequence types, C3larvinAB was demonstrated to be non-functional [9], confirming the original annotation of the corresponding toxin loci Tx7 in *P. larvae* ERIC I and TxIII in *P. larvae* ERIC II as non-functional due to multiple mutations interrupting the A and B domain genes [7].

The ability to produce toxins contributes to the virulence of *P. larvae* ERIC I strains, as shown experimentally [16]. However, the lack of toxin production in *P. larvae* ERIC II strains does not result in decreased pathogenicity or virulence; on the contrary, *P. larvae* ERIC II strains are more virulent than *P. larvae* ERIC I strains at the individual larva-level [3,5]. In other words, toxin formation increases the virulence of *P. larvae* ERIC I but is not necessary for pathogenicity or virulence of *P. larvae* ERIC II.

In this review, we will give a detailed overview of the three structurally and functionally characterized *P. larvae* AB-toxins with proven or putative ART activity, Plx1, Plx2, and C3larvinAB. We will not only recapitulate the published data on these three toxins but also put them into a wider context regarding origin, evolution, and biological relevance.

## 2. ADP-Ribosylating Toxins of *P. larvae*

### 2.1. Plx1, a Phage Born Toxin of P. larvae

The quest for virulence factors of *P. larvae* took off about ten years ago when subtractive suppression hybridization revealed genetic differences between the differentially virulent genotypes ERIC I to IV of this bacterial species [13]. Among the sequences identified as exclusively present in *P. larvae* ERIC I were two fragments that indicated the existence of a putative toxin with high similarity to the mosquitocidal toxin MTX1 of the entomopathogenic bacterium *Lysinibacillus sphaericus* [23]. Subsequent work confirmed the existence of such a toxin in the genome of *P. larvae* ERIC I and ultimately resulted in the identification and functional characterization of the toxin Plx1 as an important virulence factor for *P. larvae* ERIC I [7,16].

**Figure 1:**
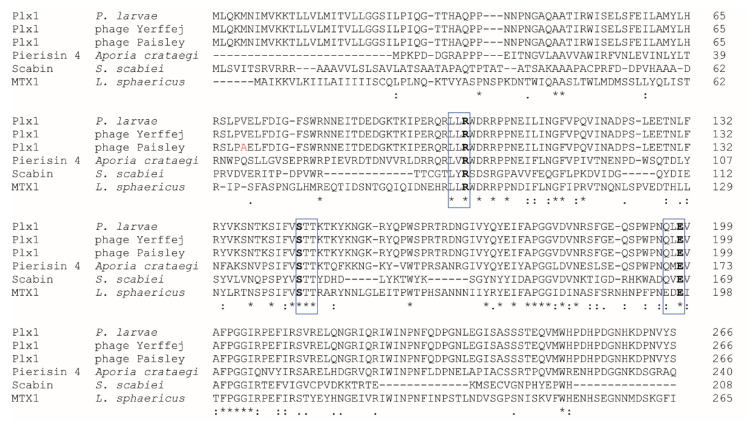
Protein sequence alignment of Plx1A and its homologs. Clustal Omega (1.2.4) multiple sequence alignment [35] of the deduced amino acid sequences of the A domains of Plx1 as found in *Paenibacillus larvae* and in the *P. larvae* bacteriophages Yerffej and Paisley compared with Pierisin 4 expressed by the butterfly *Aporia crataegi*, Scabin expressed by the plant pathogen *Streptomyces scabiei*, and the related ARTC mosquitocidal toxin MTX1 expressed by *Lysinibacillus sphaericus*. Accession numbers for these sequences are as follows: *P. larvae* Plx1, AGJ74029.1, phage Yerffej Plx1, YP_009838657.1; phage Paisley Plx1, ALA12593.1; Pierisin 4, BAH96563.1; Scabin, WP_037722833.1; MTX1, AAA22601.1. The motif L*X*R containing the conserved arginine residue, the motif STT containing the conserved serine residue, and the motif containing the conserved glutamic acid residues are boxed, the respective amino acids R-S-E are given in bold. Positions that have a single, fully conserved residue are marked by asterisks, whereas a colon or a period indicates conservation between groups of strongly similar properties or of weakly similar properties, respectively. The alanine at pos. 70 in phage Paisley Plx1 is highlighted in red.

Plx1 is a single-chain AB toxin of 975 amino acids belonging to the Cholera toxin-like ART (ARTCs) superfamily. This so-called R-S-E ART toxin superfamily is characterized by three highly conserved amino acids in the A-subunit that are all present in Plx1 (Figure 1): an arginine residue (R101) thought to be involved in maintaining the structure of the reaction pocket, a serine (S146) as part of a serine–threonine–threonine motif presumably important for the formation of the β-strand-α-helix structure and a glutamic acid (E198), which is responsible for binding the NAD and is therefore essential for ADP-ribosylation activity [24].

Plx1 is highly homologous to two toxins recently considered the enigmatic offspring from the family of ADP-ribosyltransferases [25]: MTX1 expressed by *Lysinibacillus sphaericus* [23] and the pierisins, a group of ART toxins expressed by the whites-and-yellows family *Pieridae* (Lepidoptera, Insecta) [26]. Basic Local Alignment Search Tool P (BLASTP) analysis [27,28] revealed that the overall homology of the predicted amino acid sequences of Plx1 and MTX1 was 35.58% (E-value: 1 × 10^−150^). For the pierisins, overall homology at the protein sequence level with Plx1 varied between 37.50% (Pierisin-4, *Aporia crataegi*; E-value: 8 × 10^−159^), 35.38% (Pierisin-6, *Pieris napi*; E-value: 1 × 10^−139^), and 35.05% (Pierisin-1, *Pieris rapae*; E-value: 2 × 10^−141^).

In contrast to the original assumption, the best homology of the N-terminal A-domain of Plx1 was not with MTX1 of *L. sphaericus* [11] but with the A-domains of the pierisins, a group of ART toxins expressed by the whites-and-yellows family *Pieridae* (Lepidoptera, Insecta) [29]. In particular, the essential motif for the ADP-ribosylation activity of Plx1 (196QLE198) containing the conserved glutamic acid residue is highly similar to the motif QME found in all pierisins so far but less similar to the motif (195EDE197) found in MTX1. While bacterial ADP-ribosyltransferases normally target key regulator proteins, pierisins are able to act on DNA. They were shown to ADP-ribosylate deoxyguanosine residues of DNA, thus inducing apoptosis in affected cells [30,31,32,33]. Due to the similarity of the essential motifs for transferase activity, it was suggested that Plx1 also acts on DNA [14]. However, experimental proof is lacking so far. Recently, another bacterial ART toxin, Scabin, expressed by the plant pathogen *Streptomyces scabies,* has been identified [34]. Scabin is a small (200 residues), single-domain enzyme, although a neighboring gene might encode a B-domain. The essential motif for ART activity (166QVE168) contains the glutamine residue also found in the pierisins and in Plx1. Hence, it is not surprising that Scabin was shown to target deoxyguanosine, further substantiating that ART toxins with the motif Q*X*E act on DNA and exhibit guanine specific ADP-ribosyltransferase activity.

**Figure 2:**
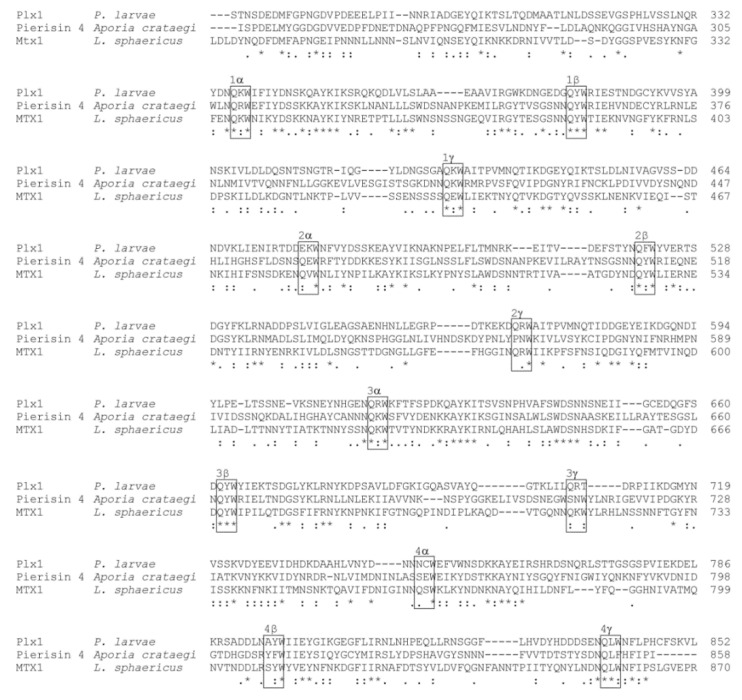
Protein sequence alignment of Plx1B and its homologs. Clustal Omega (1.2.4) multiple sequence alignment [34] of the deduced amino acid sequences of the B domains of Plx1 as found in *P. larvae* compared with Pierisin 4 expressed by the butterfly *Aporia crataegi*, and the related ARTC mosquitocidal toxin MTX1 expressed by *Lysinibacillus sphaericus*. Accession numbers for these sequences are as follows: *P. larvae* Plx1, AGJ74029.1, Pierisin 4, BAH96563.1; MTX1, AAA22601.1. The QxW motifs are boxed and the four (QxW)_3_ domains with their respective α, β, and γ QxW repeats are indicated. Positions that have a single, fully conserved residue are marked by asterisks, whereas a colon or a period indicates conservation between groups of strongly similar properties or of weakly similar properties, respectively.

The C-terminal part of Plx1 comprising the Plx1 B-subunit exhibits four ricin B-like lectin domains, which are also referred to as (QxW)_3_ domains because each domain contains three (α, β, γ) QxW motifs [16]. These lectin domains have first been described in ricin [35], an AB-toxin of *Ricinus communis* (for a recent review on ricin: [36]). Clustal Omega alignment of the binding subunits of Plx1, Pierisin-4, and MTX1 showed that the four (QxW)_3_ domains are conserved among these toxins (Figure 2). The homology of the four (QxW)_3_ domains of MTX1 with the two (QxW)_3_ domains of ricin had been demonstrated previously [37]. The lectin activity of the ricin B domain of ricin has been analyzed in detail and showed specificity for galactose and N-acetylgalactosamine, thus allowing the toxin to enter the host cell via the interaction with galactosylated receptors or other carbohydrates exposed on the host cell surface [38,39]. For Pierisin-1, high-affinity binding of its lectin domains to the glycosphingolipid receptors Gb3 and Gb4 on mammalian cells was demonstrated [40]. However, the glycoproteins or glycolipids that are used by the pierisins for cell entry into their real target cells, into lepidopteran cells, have not yet been identified. Therefore, although the role of the (QxW)_3_ domains in cell entry is well documented and we can assume that the ricin B-like lectin domains of Plx1 are involved in mediating host cell entry of the toxin, the receptors and glycosylation patterns recognized by Plx1 on the midgut cells of honey bee larvae are still unknown.

Basic Local Alignment Search Tool N (BLASTN) analysis [27,28] performed in preparing this review revealed that the entire nucleotide sequence of the Plx1 gene is also present in four temperate *P. larvae* phages, philBB_Pl23 and Yerffej, as well as Paisley and Harrison [41] with 99.97% identity and an E-value of 0.0. Consequently, Clustal Omega alignment [34] of the deduced protein sequences revealed that the four phages as well as *P. larvae* ERIC I encode a nearly identical protein of 975 aa differing in only a few amino acids resulting in 99.90% (Yerffej) to 99.79% (philBB_Pl23) protein sequence identity between the phage-encoded toxins and Plx1 (Figure 1). Plx1 expressed by the closely related phages Paisley and Harrison differs from *P. larvae* Plx1 at position 70 in the A domain (Figure 1, alanine instead of valine, highlighted in red) and phage Yerffej Plx1 differs from *P. larvae* Plx1 only at position 380 within the B domain (arginine instead of glycine). Phage philBB-Pl23 Plx1 differs from *P. larvae* Plx1 additionally at position 355 (leucine instead of glutamine). Not surprisingly, that the genomic context of *plx*1 consists of phage regions of which 22 have been found in *P. larvae* ERIC I as compared to only eight in *P. larvae* ERIC II [7]. These results suggest that the presence of Plx1 in the genome of *P. larvae* is the result of lysogenic conversion, i.e., of horizontal gene transfer from temperate phages to bacteria. Hence, Plx1 is a phage-born toxin and these four temperate phages are capable of lysogenic conversion.

Phage-borne toxin production in pathogenic bacteria has first been described in the fifties of the last century when Freeman discovered that naturally occurring avirulent strains of *Corynebacterium diphtheriae* became virulent in the presence of certain bacteriophages [42,43,44]. Subsequent studies revealed that the diphtheria toxin (DT) is only produced by lysogenic *C. diphtheriae* infected by temperate corynephages carrying the structural gene for the diphtheria toxin [45]. Once integrated, the expression of the DT gene is regulated by the host bacterium in a Fe^2+^-dependent manner [46,47]. The position of the integrated DT gene suggests that it originated from a bacterial gene that was incorporated into an ancestral corynephage through an abnormal excision event [48]. DT is not the only bacterial toxin encoded by prophages. Other examples of phage-encoded bacterial toxins are botulism toxin [49], cholera toxin [50], and Shiga toxins [51], just to name a few. However, while these phage-encoded toxins are essential for the pathogenicity and virulence of the corresponding lysogens, Plx1 only contributes to but does not determine the pathogenicity or virulence of *P. larvae* ERIC I [16]. Moreover, *P. larvae* ERIC II strains, which typically lack Plx1, are more virulent at the larval level than *P. larvae* ERIC I strains [5]. Knowing that Plx1 is phage-born, it makes sense to screen *P. larvae* strain libraries to capture the true frequency of Plx1 in the different *P. larvae* genotypes. This will help to elucidate the preferences of these temperate phages for certain genotypes of *P. larvae* and to understand the interaction between them and *P. larvae* ERIC I and ERIC II.

Interestingly, it has been suggested that the pierisins may have been derived from bacteria via horizontal gene transfer [52,53]. In addition to the genomic organization of the pierisin-1 gene in the genome of *Pieris rapae* [52], the existence of the pierisin-homolog Plx1 expressed by the entomopathogenic bacterium *P. larvae* [16] was another argument in favor of the hypothesis that a bacterial DNA ADP-ribosylating toxin could be the ancestor of the pierisins [53]. Now that we know that Plx1 is a phage-borne toxin, the pierisins could turn out to represent a toxin family that has its roots in the virus world before it was transferred to certain butterflies via bacteria. In addition to this interesting question regarding the evolution of the pierisins, the evolution of Plx1 is also worth being studied. The presence of Plx1 in genetically unrelated *P. larvae* phages [54] poses the question of whether it was incorporated from a *P. larvae* ancestor into an ancestral *P. larvae* phage through an abnormal excision event and is now reintroduced into *P. larvae* via lysogenic conversion. Further studies are also necessary to elucidate the stability and regulation of the phage-born *plx*1-gene, which enables *P. larvae* to use Plx1 as a weapon against bee larvae.

### 2.2. C3-Like Toxins with B-Subunits

**Figure 3:**
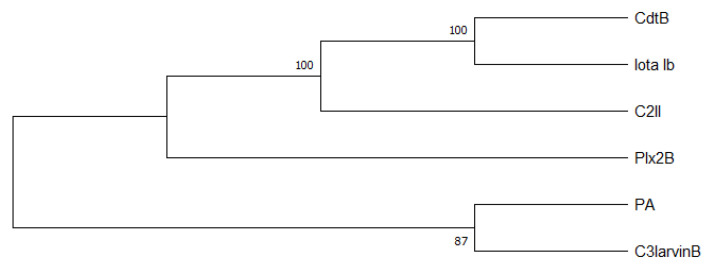
Phylogenetic tree created with the neighbor-joining method (MEGA X Version 10.1.8) [62,63]. Shown is the optimal tree with the sum of branch length = 2.93596097. The results of the bootstrap test (500 replicates) are shown [%]. Furthermore, the Poisson correction method calculating the evolutionary distances was used. Ambiguous positions were removed for each sequence pair (pairwise deletion option).

C3-like toxins normally are characterized as single domain toxins that act on cells without a B subunit partner. *P. larvae* interestingly has two AB toxins, Plx2 and C3larvinAB, which have a B subunit although their A domains have clearly been identified as members of the C3-like toxin family [9,16]. The A subunits of the *P. larvae* binary C3-like toxins, Plx2A and C3larvinA [16,22,55], possess conserved motives and conserved residues typical for C3-like toxins: α-3 motif, catalytic Arg, STS motif, PN loop, ARTT loop, and catalytic Q*X*E motif (as reviewed by [56]). The B subunits, Plx2B and C3larvinB [9,16], both comprise a putative binary_toxB domain, which is usually characteristic for the B subunits from other AB toxins like C2 toxin component II (C2II) from *Clostridium botulinum*, binary toxin B (CdtB) from *C. difficile*, iota toxin from *C. perfringens*, and anthrax toxin protective antigen (PA) component from *Bacillus anthracis* [57,58,59,60,61]. To visualize the relationship of C3larvinB and Plx2B with C2II, CdtB, Iota Ib, and PA, we constructed a phylogenetic tree using the neighbor-joining method [62] and showed that C3larvinB is closely related to PA, whereas Plx2B is more closely related to CdtB, Iota Ib, and C2II (Figure 3). The B subunits are known for a rather high structural similarity, whereas the similarity on protein sequence level of most B subunits is between about 31% and 44% (Table 1). Furthermore, Plx2B and C3larvinB contain a putative carbohydrate-binding domain, which might be involved in binding of the toxin complex to glycoproteins and glycolipids on the honey bee host cell surface [9,16].

**Figure 4:**
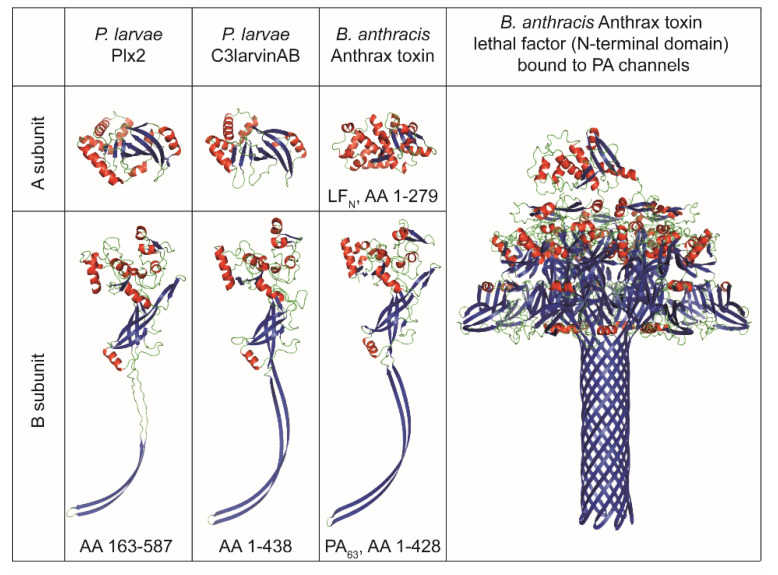
Structural comparison of the *P. larvae* binary C3-like AB toxins to *B. anthracis* anthrax toxin. Protein structure visualization was done with PyMOL Molecular Graphics System (Schrödinger, LLC, Mannheim, Germany). The structural visualizations are based on Plx2A X-ray structure, PDB acc. no. 5URP [52]; Plx2B structural prediction, GenBank acc. no. AGJ74030.1 [14]; C3larvinA structural prediction, GenBank acc. no. QDD55731.1 [9]; C3larvinB structural prediction, GenBank acc. no. QDD55730.1 [9]; Anthrax toxin lethal factor N-terminal domain (LF_N_), PDB acc. no. 1PWQ [78]; Anthrax toxin activated protective antigen 63 kb-fragment (PA_63_), PDB acc. no. 3J9C [66]; *B. anthracis* Anthrax toxin lethal factor (N-terminal domain) bound to PA channels cryo-EM structure PDB acc. no. 6PSN [73]. Structural predictions were carried out by the use of the protein modeling homology server CPHmodels 3.2 [79]. The template structure for the structural predictions of Plx2B and C3larvinB were both based on anthrax toxin PA, PDB acc. no. 3J9C. The template structure for the structural prediction of C3larvinA was based on C3larvin, PDB acc. no. 4TR5. Color code: α-helices, red; β-sheets, blue.

A structural comparison of the two *P. larvae* binary AB toxins to *B. anthracis* anthrax toxin shows a striking similarity of the activated B subunits of all three toxins (Figure 4). Anthrax toxin is a binary toxin composed of the binding subunit protective antigen (PA), which can interact with two possible enzymatically active A subunits, lethal factor (LF) and edema factor (EF). PA is enzymatically activated via furin cleavage, and thereupon, the 63 kDa-sized monomers (PA63) can form a hepta- or octameric translocase channel [64,65]. The PA63 monomers have an extended β-hairpin structure, which is also found in Plx2B and C3larvinB (Figure 4). The PA channel or pore has a “flower-on-a-stem” architecture and translocates one of the A subunits, either lethal factor (LF) or edema factor (EF), into the cell [66]. A similar channel structure has recently also been identified for the binding components iota toxin Ib of *C. perfringens* and CdtB of *C. difficile* [67,68]. If Plx2B or C3larvinB also form such channels still needs to be investigated. LF is a 91 kDa-sized protease targeting the mitogen-activated protein kinase (MAPK) pathway [69], while EF is an 89 kDa-sized adenylate cyclase [70]. The N-termini of LF (LFN) and EF are highly homologous. Specifically, an N-terminal α-helix is involved in PA-binding at the α-clamps, which are clefts formed at the surface of PA between two PA subunits and bind to α helices non-specifically [71,72,73].

The A subunits of the about 25 kDa-sized C3-like A subunits, Plx2A and C3larvinA, are structurally homologous among each other, which is very characteristic of the C3-like toxin group [55,56], but differ structurally from LFN (Figure 4). However, the importance of an N-terminal α-helix has also been demonstrated for C3-like toxins. The N-terminal α-helix-1 was shown to be important for cell entry of C3bot1, C3lim, and C3larvinA [9,21,22,74]. An in silico sequence-structure-function comparison study suggested that conserved motifs of the N-terminus influence catalytic activity, inter-domain stability, and binding and/or translocation [75]. In this study, conserved residues were found in the N-terminal region within and around α-helix-1 of Plx2A, C3larvinA_trunc_, and different C2- and C3-like toxins [75]. The conserved residues presumably belong to different motifs involved in protein activity, protein stability, and binding [75]. The effect on structure and function of the N-terminal α-helix-1 region of C3larvinA was experimentally further characterized with deletion and substitution variants, and an influence on protein stability and enzymatic activity were confirmed [76]. In C2-like toxins, the N-terminal part has been shown to be responsible for interaction with the B subunit partner [77]. The interaction between the A and B subunits of Plx2 and C3larvin and the cell entry mechanisms into the larval honey bee cell remains open for investigations in future studies. As Plx2 and C3larvinAB share an A subunit homologous to C3-like toxins but also possess a B subunit partner with similarity to C2-like toxins, they might be an evolutionary connection between these two toxin classes and deserve special attention.

#### 2.2.1. Binary C3-Like Toxin Plx2

*P. larvae* toxin 2, Plx2, is an important virulence factor of *P. larvae* [16]. It is a binary AB toxin that consists of a catalytically active A subunit, Plx2A, and a B subunit, Plx2B. Plx2B mediates the binding of Plx2A to the target cell and the uptake of the toxin into the cell. The subunits of Plx2 are encoded by two separate genes.

Plx2A has typical properties of C3 exoenzymes. The homology to other AB toxins is further supported by the presence of characteristic motifs. The conserved motifs of the A domain of Plx2 are crucial for substrate binding and catalyze the enzymatic reaction. The 166QxE168 motif in the active site is typical for mono ADP-ribosyltransferases whose substrates are Rho proteins [56]. All these in silico predictions of the protein properties based on the genomic level were confirmed for Plx2A in further analyses [55]. For this purpose, different variants of the protein were specified, which differed from the wild type in the various conserved domains. In addition, crystal structure analyses of Plx2A were carried out, which clearly confirmed the homology to other C3 toxins once again. Thus, it can now be said without a doubt that Plx2A belongs to the C3 mono-ADP ribosylating toxins, uses NAD+ as the substrate from which an ADP-ribose residue is transferred to the target RhoA. On the basis of in silico analyses as well as by molecular and protein biochemical studies, the classification of Plx2A was clearly established. Nevertheless, Plx2 is a special case within the toxin families since no B domains have been described for the previously known C3 toxins.

The B subunit of Plx2, on the other hand, is homologous to B subunits of C2 toxins, such as C2 actin ADP-ribosylating binary toxin from *Clostridium botulinum* [57] and the toxin CDT from *Clostridium difficile* [80] and presents motifs that presumably can cause binding to carbohydrates [81]. Plx2 with its C3 homologous A domain and the B domain, which is homologous to C2 toxins, thus represents the intersection between the C2 and C3 toxins.

It has been shown that Plx2A has ADP-ribosyltransferase activity and that it targets RhoA in the host cell [55]. RhoA is a small GTPase protein that belongs to the Ras superfamily [82]. The members of this family are mostly involved in the control of different aspects of actin remodeling, including cytokinesis [83,84,85]. Cell culture experiments have shown that Plx2A differentially affected mammalian and insect cells. In mouse macrophages, Plx2A induced reorganization of the cytoskeleton resulting in changes in cell morphology and the formation of filopodia-like protrusions [55]. In contrast, in Plx2A-treated insect cells (*Trichoplusia ni*, Tn5; *Spodoptera frugiperda*, Sf9), the actin cytoskeleton remained unaltered but increased vacuolization, and the formation of bi-nucleated cells were found, indicating that in insect cells, Plx2A has an inhibitory effect on cytokinesis [55].

The different effects of Plx2A on different cell types illustrate the different modes of action of RhoA. It is known that the RhoA activity is mainly associated with the regulation of the cytoskeleton, which involves, for example, the formation of actin stress fibers [86,87]. Regulatory functions in cell development and transcription are also described for RhoA [88]. It has even been described in invertebrates that RhoA GTPases are involved in the regulation of innate immunity. In the shrimp *Marsupenaeus japonicas* RhoA has been shown to have an anti-bacterial function due to its involvement in integrin-mediated phagocytosis of bacteria by hemocytes, which protects the host from microbial infection [89]. It is possible that RhoA has a similar function in honey bee hemocytes. If this were the case, in addition to acting as a toxin for larval midgut epithelial cells, Plx2 could disrupt phagocytosis and thus inhibit the innate response of the larvae to infection with *P. larvae*. Further studies are needed to analyze this aspect of Plx2 activity. In any event, the fact that Plx2A differentially affected mammalian and insect cells make it a promising new tool for studying the effects of RhoA in invertebrate and vertebrate cells.

**Figure 5:**
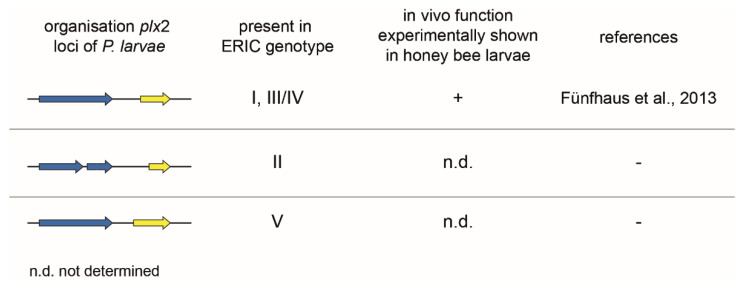
Representation of the *plx*2 toxin loci in *P. larvae* ERIC I–V. The *plx*2 locus of *P. larvae* ERIC I shows the same genomic organization as the toxin loci of ERIC III and IV. The activity of the protein, as well as an influence of Plx2 on the virulence of *P. larvae* ERIC I, has already been demonstrated [16,55]. It can therefore be assumed that Plx2 of ERIC III and IV will have the same impact. The ERIC II *plx*2B locus, on the other hand, is fragmented and the *plx*2A is significantly shortened. Here it can be assumed that the functionality of the toxin is no longer given. *P. larvae* ERIC V, however, has an N-terminally prolonged *plx*2A locus, which could lead to an increased toxic activity of the protein and increased virulence of the *P. larvae* strain DSM 106053. The arrows symbolize the open reading frames (ORFs) with the following color code: blue, B subunit; yellow, A subunit.

The genomes of different *P. larvae* isolates from five different *P. larvae* genotypes (ERIC I–V) were analyzed for the presence of the Plx2 locus. This toxin locus can be found in strains of the genotypes ERIC I, III/IV, and V (Figure 5). In *P. larvae*, ERIC II Plx2 is only present in fragments and is therefore probably no longer functional [16]. On the other hand, the *P. larvae* strain DSM 106052 (ERIC V) has a 126 bp longer N-terminal Plx2A sequence. The importance of the N-terminus for cell entry, respectively interaction with a potential B subunit partner, and also structure and catalytic activity has been demonstrated in former studies with C3-like and C2-like toxins [21,74,75,76,90]. Therefore, it might be an interesting subject for future in vitro and in vivo studies to analyze whether the longer N-terminus of Plx2A in *P. larvae* ERIC V mediates a higher activity or higher mortality in honey bee larvae.

Plx2 is a protein that has been shown to be expressed by *P. larvae* ERIC I. Exposure bioassays with corresponding gene inactivation mutants compared to the wild type strain have shown that Plx2 is an important virulence factor of *P. larvae* ERIC I [16]. Due to the sequence identity, the same effect is also expected for the *P. larvae* genotypes ERIC III and IV. Since these genes are only fragmented in strains of the genotype ERIC II, this toxin is probably not functional there. The Plx2 activity of DSM 106052 (*P. larvae* ERIC V) with the extended N-terminus of the A subunit has yet to be determined.

#### 2.2.2. Binary C3-Like Toxin C3larvinAB

**Figure 6:**
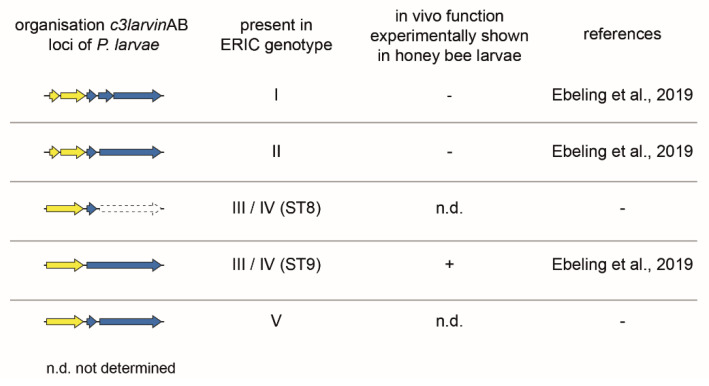
Genomic organization of the *c3larvin*AB gene loci of *P. larvae* ERIC I-V. The *c3larvin*AB gene locus was examined experimentally with *P. larvae* knockout mutants in exposure bioassays in honey bee larvae. Therein, C3larvinAB was only confirmed as a virulence factor in *P. larvae* ERIC III (ST9) [9]. The arrows symbolize the open reading frames (ORFs) with the following color code: blue, B subunit; yellow, A subunit.

C3larvinAB (formerly named Tx7 in ERIC I and TxIII in ERIC II) was first identified as a putative binary AB toxin in *P. larvae* ERIC I and ERIC II in a whole-genome sequence comparative study [7]. However, because of the disruption of the ORF due to a stop mutation, the toxin gene locus of the A subunit lacks the N-terminal part encoding a signal sequence and α-helix 1 (Figure 6). The B subunit toxin gene locus was disrupted by one (ERIC II) or two (ERIC I) stop mutations and thus was declared as non-functional. The C3larvin AB toxin as a whole was supposed to be non-functional as AB toxins require both subunits to act as functional toxins [7].

The truncated A subunit was recombinantly expressed in *E. coli* and identified as typical C3-like toxin, which ADP-ribosylates RhoA as a cellular target [21]. However, the recombinant truncated C3larvin was unable to enter murine macrophage cells as was formerly observed for the C3-like toxins C3bot1 and C3lim [74]. Fahrer and co-workers [74] suggested that the N-terminal α-helices might be important for the cell entry of C3bot1 and C3lim. As the truncated C3larvin of *P. larvae* ERIC I and II lacks the N-terminal α-helix 1, a chimera was constructed with the equivalent N-terminal part of C3bot1. The chimera was able to enter the macrophages and cause morphological changes [21], reinforcing both the relevance of α-helix 1 for the functionality of C3-like toxins [74] and the initial classification of this toxin gene locus in *P. larvae* ERIC I and ERIC II as non-functional [7]. The latter was further supported by testing gene inactivation mutants of *P. larvae* ERIC I and ERIC II, which were no longer able to express C3larvinA in exposure bioassays with honey bee larvae: The absence of C3larvinA expression had no effect on the mortality of the honey bee larvae, hence the virulence of *P. larvae* [9]. In the same study, one *P. larvae* strain (11-8051) from ST9 of genotype ERIC III/IV was identified, which comprised a complete C3larvinAB toxin locus consisting of a non-truncated A subunit of C3larvin (C3larvinA) and also a full-length B subunit (C3larvinB) (Figure 6). Interestingly, while the knockout of C3larvinA did not cause any effect on larval mortality, the knockout of C3larvinB resulted in reduced larval mortality when compared to the wildtype strain [9]. Consequently, as the A and B subunits can only function together, this toxin version was declared a functional virulence factor of *P. larvae* ST9 (strain 11-8051). The further examination of genomic data also identified intact C3larvinA loci in *P. larvae* ERIC III and IV (ST8) and also in ERIC V (ST25) [8,9]. However, since C3larvinB is incomplete and interrupted in these genotypes (Figure 6), these strains were not included in any functional assays so far.

In a further study, the full-length C3larvinA from *P. larvae* strain 11-8051 (ST9) was recombinantly expressed and enzymatically characterized, and here, cell entry in murine macrophages was successful and morphological changes were induced due to an effect of C3lavinA on the actin cytoskeleton [22]. Furthermore, the recombinant, non-truncated C3larvinA, as expressed by *P. larvae* strain 11-8051 (ST9), was also able to enter insect cells and caused the same effect as the other C3-like binary AB toxin from *P. larvae*, Plx2A, with bi-nucleated cells indicating interference with cytokinesis [9,22,55]. Both C3-like toxins of *P. larvae* targeting host cell RhoA interfere with the cytoskeleton in mammalian cells but with cytokinesis in insect cells, indicating differential roles for RhoA GTPases in mammals versus insects and recommending these *P. larvae* toxins as tools for studying these differences.

## 3. Outlook

So far, three P. *larvae* toxins have been biochemically and/or functionally characterized in detail. However, other putative toxin loci are known [7]. After assessing the individual loci, it can be assumed that the *plx*3–*plx*5 loci are complete. All other putative toxin loci of *P. larvae* appear to be fragmented and the corresponding proteins are therefore probably not functional. Plx3 is similar to Plx1. It is also a single-chain AB toxin with a putative active site, which also has a QLE sequence typical for ADP-ribosyltransferases acting on DNA. Plx4 and Plx5, however, have separate A and B subunits like Plx2. The A subunit shows similarity to ADP-ribosyltransferases of *B. cereus* and *B. thuringiensis,* and the B subunit is similar to the B subunits of C2 toxins of *C. botulinum* and CDT of *C. difficile*. However, the B subunit of Plx5 again appears to be interrupted [7]. The functionality of the individual toxins should be investigated in further studies in order to advance beyond mere assumptions about the properties of the various toxins on the basis of the available genomic sequences of *P. larvae*.

In addition, it is conceivable that the toxin genes are organized in operon structures, but it is striking that the gene order is different for the binary toxins Plx2 (Figure 5) and C3larvinAB (Figure 6). For operons, as functional units of bacterial gene expression, the gene order is crucial because it has a direct influence on the assembly order of protein complexes [91]. These considerations can be a starting point for further analyses.

## Figures and Tables

**Table 1:** The percentage similarity of B subunit amino acid sequences calculated with BLASTp.

	Plx2B	C3larvinB	PA	Ib	C2II	CdtB
**Plx2B**	-	40.36%	31.24%	37.87%	34.89%	38.54%
**C3larvinB**	40.36%	-	39.45%	36.55%	35.11%	39.69%
**PA**	31.24%	39.45%	-	35.86%	35.51%	34.46%
**Ib**	37.87%	36.55%	35.86%	-	43.43%	79.93%
**C2II**	34.89%	35.11%	35.51%	43.43%	-	44.76%
**CdtB**	38.54%	39.69%	34.46%	79.93%	44.76%	-

## Data Availability

No new data were created or analyzed in this study. Data sharing is not applicable to this article.
